# Arterial-only anastomosis for fingertip avulsion amputations: survival rates and functional outcomes in a retrospective study of 194 digits

**DOI:** 10.1186/s12893-026-03692-8

**Published:** 2026-03-26

**Authors:** Zefu Weng, Ailifeire Ainiwaer, Wenquan Ding

**Affiliations:** 1 Health Science Center, Ningbo University, Ningbo, Zhejiang China; 2 Ningbo Clinical Research Center for Orthopedics, Sports Medicine & Rehabilitation, Department of Hand Surgery, Ningbo No.6 Hospital, Ningbo, Zhejiang China

**Keywords:** Fingertip, Amputation, Replantation, Anastomosis, Microsurgery, Ischaemia, Neovascularisation, Haemostasis, Haemorrhage, Thrombosis

## Abstract

**Background:**

While not all fingertip avulsion amputations progress to necrosis after replantation, studies that examined survival rates of replanted digits or key influencing outcomes in fingertip avulsion amputations are lacking. Therefore, we investigated the incidence of postoperative vascular crises, survival rates of replanted digits, functional outcomes, and factors influencing outcomes in patients with fingertip avulsion amputations treated with arterial-only anastomosis.

**Methods:**

A retrospective study was conducted on completely amputated fingertips undergoing attempted replantation at Ningbo No. 6 Hospital between March 2017 and December 2024. Only digital arteries or arterial arch branches were identifiable for anastomosis; venous anastomosis was not feasible. According to our inclusion criteria, all 194 digits included in the final analysis had successful restoration of arterial blood flow confirmed intraoperatively. Group 1 included 84 patients with 98 avulsion amputations, and Group 2 included 82 patients with 96 clean-cut amputations. Postoperative vascular crises were recorded, and management—anticoagulation, thrombolysis, or intermittent bleeding—was tailored to arterial or venous compromise. Survival rates of replanted digits were assessed, and functional outcomes of surviving fingertips were compared between the groups.

**Results:**

This study evaluated outcomes in a cohort of 194 digits. Arterial crises occurred in 28 digits (22 in Group 1; 6 in Group 2), whereas venous crises occurred in 86 digits (44 in Group 1; 42 in Group 2). In 22 digits (12 in Group 1; 10 in Group 2), venous crises preceded arterial crises. A total of 65 digits developed necrosis (42 in Group 1; 23 in Group 2). The overall survival rate was 66.5% (57.1% in Group 1; 76.0% in Group 2, *p* = 0.006). Functional scores for surviving digits were excellent in both groups.

**Conclusions:**

Despite a lower survival rate than conventional arterial–venous anastomosis, arterial-only replantation for fingertip avulsion amputations achieved a survival rate exceeding 50% in this study. The absence of tendon and joint injury at this level contributed to acceptable functional and cosmetic outcomes among surviving digits. Therefore, arterial-only replantation represents a feasible and valuable alternative for complex fingertip avulsions where venous repair is not possible, offering a worthwhile attempt at digital salvage in selected cases.

**Supplementary Information:**

The online version contains supplementary material available at 10.1186/s12893-026-03692-8.

## Background

**Fig. 1:**
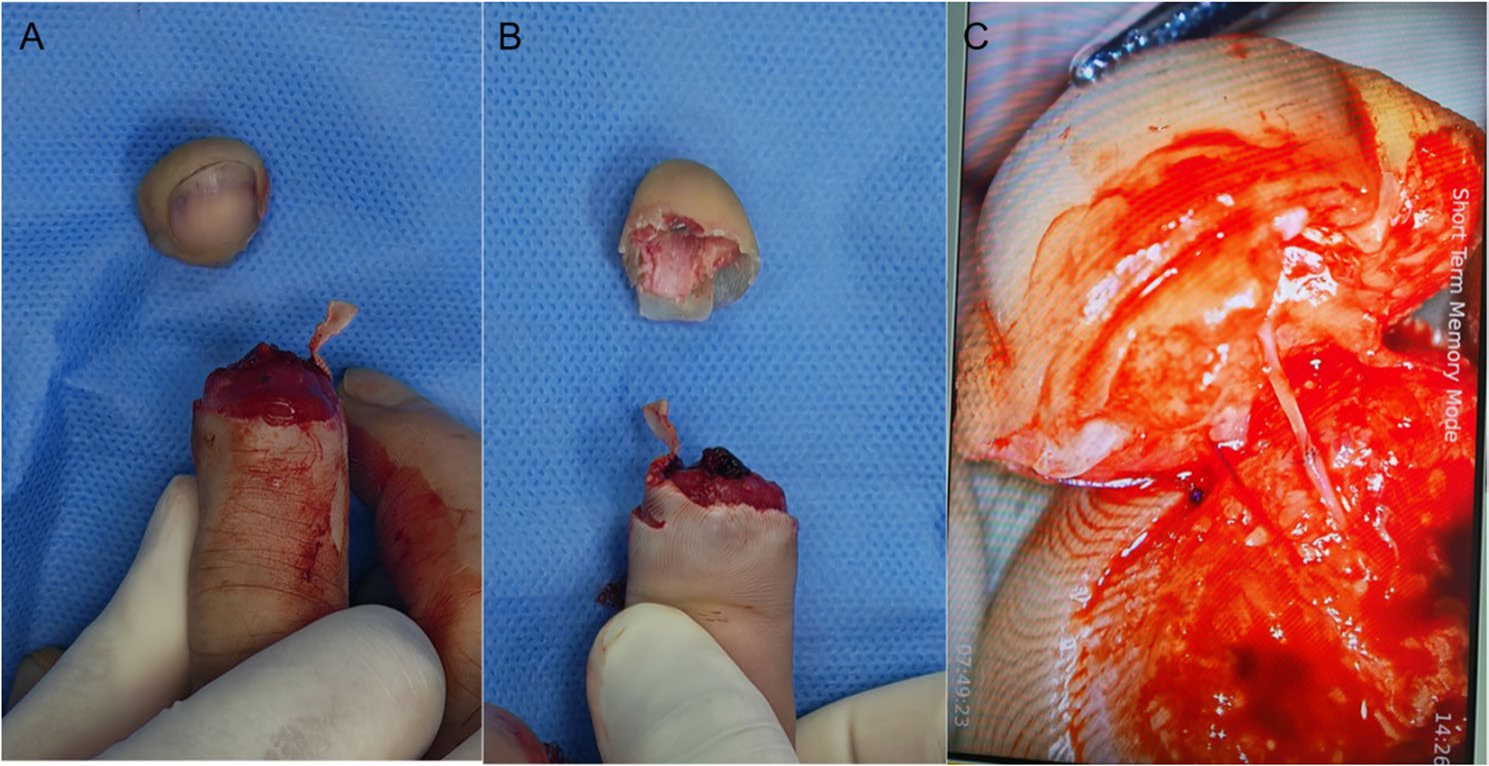
Fingertip amputation case: arterial anastomosis was possible, but no suitable veins were available for anastomosis. **A** and **B** Tamai type I amputation. **C** Arterial anastomosis was performed during replantation, but no suitable veins for anastomosis could be identified

Fingertip amputations are common injuries that occur in occupational environments and daily activities. The primary treatment option for these injuries is stump revision and replantation. However, stump revision can compromise the function and appearance of the injured digit. Advances in microsurgical techniques, expanding knowledge of fingertip microanatomy, and increasing functional demands have made replantation of fingertip avulsion amputations—once considered contraindicated—a routine surgical procedure [1]. The fingertip, located distal to the nail root, has unique anatomical features characterised by few terminal vessels and small-calibre lumens. Digital arteries within the amputated fingertip can sometimes be identified and anastomosed under a microscope. However, fingertip veins are extremely small, lie superficially beneath the volar pulp dermis, and are frequently severely damaged during digital injuries. Consequently, suitable veins for anastomosis are rarely available (Fig. 1). Replantation with arterial-only anastomosis often leads to arteriovenous circulatory disorders, postoperative venous crises, and eventual necrosis of replanted digits, leading to replantation failure [2].

According to the Tamai classification [3], distal phalanx amputations are characterised into two types: type I, extending from the proximal edge of the lunula to the fingertip, and type II, extending from the distal interphalangeal joint to the lunula. Most fingertip amputations are Tamai type I, for which replantation typically relies on arterial-only anastomosis. This technique does not involve establishing venous drainage, resulting in the absence of physiological perfusion. Spontaneous venous drainage can be established relatively quickly because of the small tissue volume in the fingertip, its relative tolerance to low perfusion, and rich vascular supply. Postoperative adjuncts, including small incisions to promote bleeding or nail bed bleeding, are often used to facilitate venous drainage [4]. Medical leeches have also been used to promote venous drainage; however, their associated survival rate is markedly lower than those achieved with venous anastomosis [5].

Our clinical observations indicate that not all fingertip avulsion amputations progress to necrosis after replantation. However, our literature identified no studies that examined survival rates of replanted digits or key influencing outcomes in fingertip avulsion amputations. Therefore, we conducted a retrospective study of patients with fingertip avulsion amputations to investigate the incidence of postoperative vascular crises, the survival rate of replanted digits, key prognostic factors, and functional outcomes. We hypothesised that the rates of vascular complications and survival would be different between avulsion-type amputations and clean-cut amputations owing to more extensive soft tissue and vascular damage with the former, even when both are managed with the same arterial-only strategy.

## Methods

This retrospective study investigating the incidence of postoperative vascular crises, survival rate of replanted digits, key prognostic factors, and functional outcomes of fingertip amputations was approved by the Ethics Committee of Ningbo No. 6 Hospital. In this study, we analysed patients with complete fingertip amputations who underwent attempted replantation at Ningbo No. 6 Hospital, between March 2017 and December 2024. The inclusion criteria were as follows: (1) during replantation, only digital arteries or branches of the arterial arch were identifiable and suitable for anastomosis, and venous anastomosis was not feasible; (2) arterial blood flow was successfully restored in the replanted digits during the procedure. The exclusion criteria were (1) absence of a suitable artery for anastomosis in the amputated digit during replantation and (2) extensive subcutaneous vascular network injury caused by crushing, resulting in persistent absence of fingertip skin perfusion after arterial anastomosis.

**Fig. 2:**
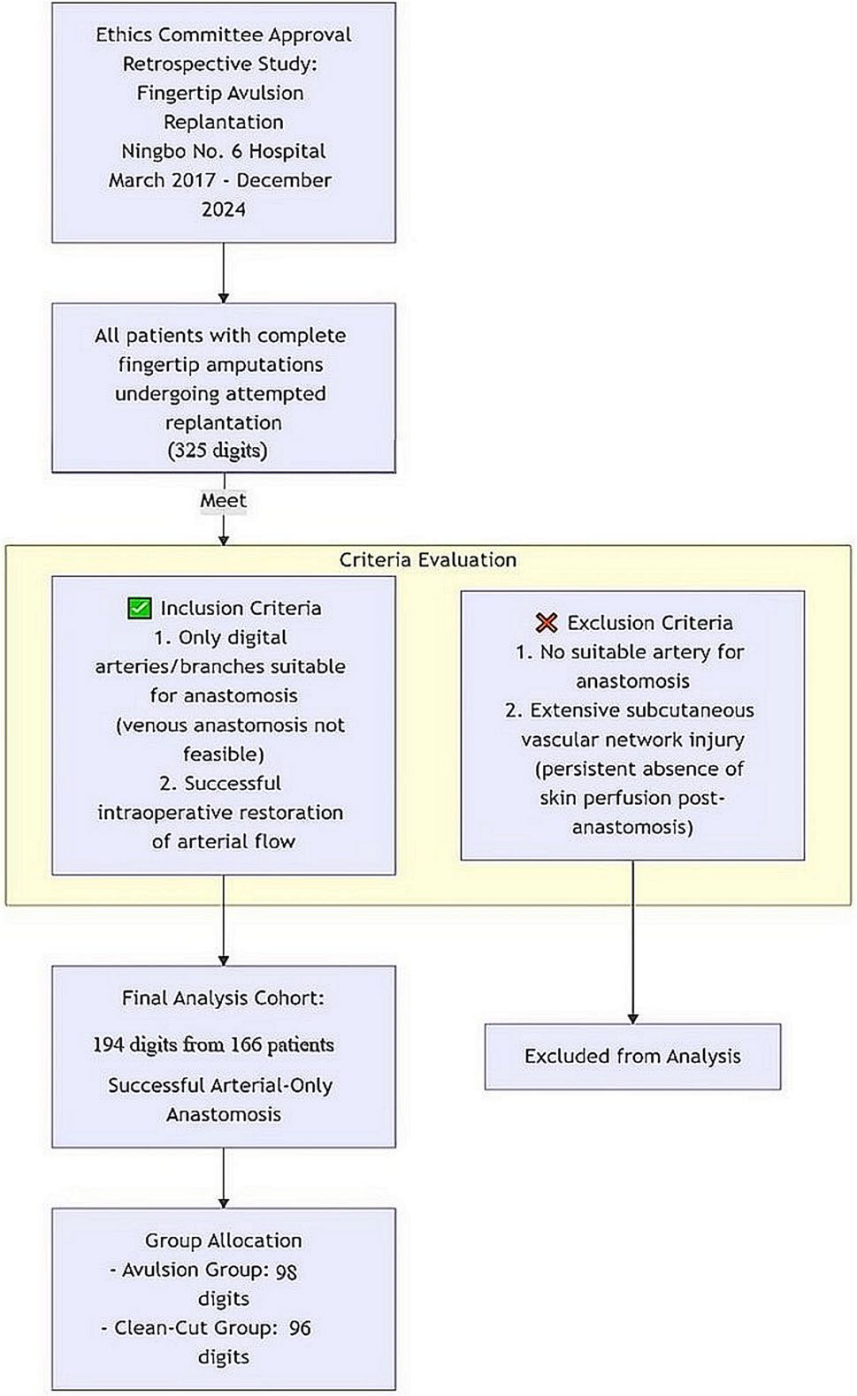
Patient and Digit Selection Flow Diagram

According to our inclusion criteria, all 194 digits included in the final analysis had successful restoration of arterial blood flow confirmed intraoperatively. Among these cases, 84 patients with 98 fingertip avulsion amputations were assigned to Group 1, whereas 82 patients with 96 clean-cut amputations were assigned to Group 2 (Fig. 2). The baseline characteristics of patients and injuries were presented in Table 1.

**Table 1:** Baseline characteristics of patients and injuries

		Group 1	Group 2	p-value
Patients	Age, median (interquartile range)	41 (30,48)	42 (28,52)	0.226 (95%CI: 0.216–0.233)
	Sex	male 62; female 22	male 60; female 22	1.000
	Smoking	Yes 29; No 55	Yes 34; No 48	0.751
	Diabetes	Yes 13; No 71	Yes 14; No 68	0.208
	Dominant hand involvement	Yes 36; No 48	Yes 38; No 44	0.439
	Multiple digit injuries	Yes 13; No 71	Yes 9; No 73	0.175
Digits	Injured digit	Thumb 18; index finger 22; long finger 39; ring finger 18; little finger 1	Thumb 28; index finger 24; long finger 26; ring finger 14; little finger 4	
	Tamai level	I 95; II 3	I 94; II 2	1.000
	Ischaemia time(h), median (interquartile range)	4.5 (4,5)	4.0 (3,5)	0.508 (95%CI: 0.498–0.517)

Postoperatively, patients in both groups were admitted to a specialised replantation ward for vascular monitoring and received standard infection prophylaxis, antispasmodic and anticoagulant therapies, and infrared lamp warming. They were asked to maintain strict bed rest with the head of the bed elevated to approximately 15° and the affected limb positioned at the side of the body. No external immobilization device was applied.

### Definitions of postoperative vascular crises

Arterial crisis was diagnosed on the basis of the following clinical signs observed in the replanted digit: pallor or a waxy white colour; significant decrease in tissue turgor (flabbiness); a marked drop in skin temperature (typically > 2 °C compared with the temperature of an adjacent uninjured digit); significantly prolonged (> 3 s) or absent capillary refill; and scant, bright red bleeding from the pulp incision or nail bed.

Venous crisis was diagnosed on the basis of the following clinical signs: dark purple or cyanotic discolouration; increased tissue turgor (tension and swelling); initially rapid (< 1 s) capillary refill that later became absent; and active, dark red bleeding from the pulp incision or nail bed.

### Anticoagulation and antispasmodic regimens

The patients were prescribed anticoagulation and antispasmodic regimens for 7 days postoperatively or until vascular stability was achieved; the former included low-molecular-weight heparin (Clexane; 4250 IU IH QD) and the latter included papaverine (30 mg IM Q8H).

### Thrombolysis protocol

When arterial or venous crisis persisted despite the anticoagulation and antispasmodic therapies, thrombolysis was indicated. Exploratory thrombolysis was performed by intravenous infusion of urokinase at doses ranging from 100,000 to 300,000 units.

### Intermittent bleeding protocol

The intermittent bleeding protocol was implemented as follows. A superficial, coronally oriented incision was made on the palmar aspect of the fingertip pulp and extended to the dermis with careful preservation of the underlying subcutaneous tissue. This procedure was repeated every 2 h. Bleeding was initiated by gentle stroking of the incision site with a needle dipped in heparin solution. Bleeding and patency were maintained by wiping the site five times with a heparin-soaked pledget.

### Monitoring schedule

All patients were specifically monitored for vascular complications immediately after surgery until vascular stability was achieved or necrosis was confirmed. Intensive monitoring involved hourly assessments of colour, turgor, skin temperature, and capillary refill for the first 7 postoperative days, which covers the period of highest risk for vascular compromise. Thereafter, monitoring was continued at less frequent intervals until discharge. The pharmacological interventions—anticoagulation, antispasmodic, and thrombolytic therapies—were exclusively utilised for adult patients. In contrast, the mechanical intervention of intermittent fingertip bleeding was employed for patients of all ages.

The incidence of vascular crises and the survival rates of replanted digits were recorded for both groups. Surviving replanted digits underwent regular follow-up. At 6 months postoperatively, functional outcomes were assessed using the provisional criteria for digit replantation assessment established by the Chinese Society of Hand Surgery, of the Chinese Medical Association (Additional File 1). The evaluation included total active motion of finger joints, activities of daily living scores, fingertip sensory recovery, blood circulation status, appearance, and return-to-work status. Sensory recovery and cold intolerance in the fingertip were specifically assessed as part of the circulation and sensation domains of the functional score.

### Statistical analysis

Categorical variables were compared using the chi-square test or Fisher’s exact test. The normality of all continuous variables was assessed using the Shapiro–Wilk test. For data that followed a normal distribution, the results are presented as mean ± standard deviation, and group comparisons were made using the independent samples t‑test. For data that did not follow a normal distribution, the results are expressed as median (interquartile range), and group comparisons were performed using the Mann–Whitney U test.

## Results

All outcome data were analysed and are reported according to the number of replanted digits. Digits were included in the final analysis only if intraoperative restoration of arterial flow was achieved (*n* = 194). Postoperative arterial crises occurred in 28 digits (22 in Group 1 and 6 in Group 2), whereas venous crises developed in 86 digits (44 in Group 1 and 42 in Group 2). In 22 digits (12 in Group 1 and 10 in Group 2), venous crises occurred before arterial crises. Given the low patency rate of re-anastomosis for the fingertip vessels (because of insufficient vessel length after resection of the thrombosed segment and the limited applicability of venous grafts in fingertip replantation), salvage re-anastomosis was not attempted for any replanted digit that developed vascular compromise. Ultimately, necrosis developed in 65 digits (42 in Group 1 and 23 in Group 2). For the digits that progressed to necrosis, revision amputation was performed once the demarcation of necrosis became clear. The overall survival rate was 66.5% (57.1% in Group 1 and. 76.0% in Group 2, *p* = 0.006). According to the provisional criteria for digit replantation assessment established by the Chinese Society of Hand Surgery, the Chinese Medical Association, the median (interquartile range) functional score of surviving replanted digits was 89.5 (87, 93.75) (*n* = 56) in Group 1 and 90 (87, 96) (*n* = 73) in Group 2 (*p* = 0.100; Monte Carlo two-tailed *p* = 0.096; 95% CI: 0.090 to 0.102). Both groups demonstrated excellent functional ratings.

**Fig. 3:**
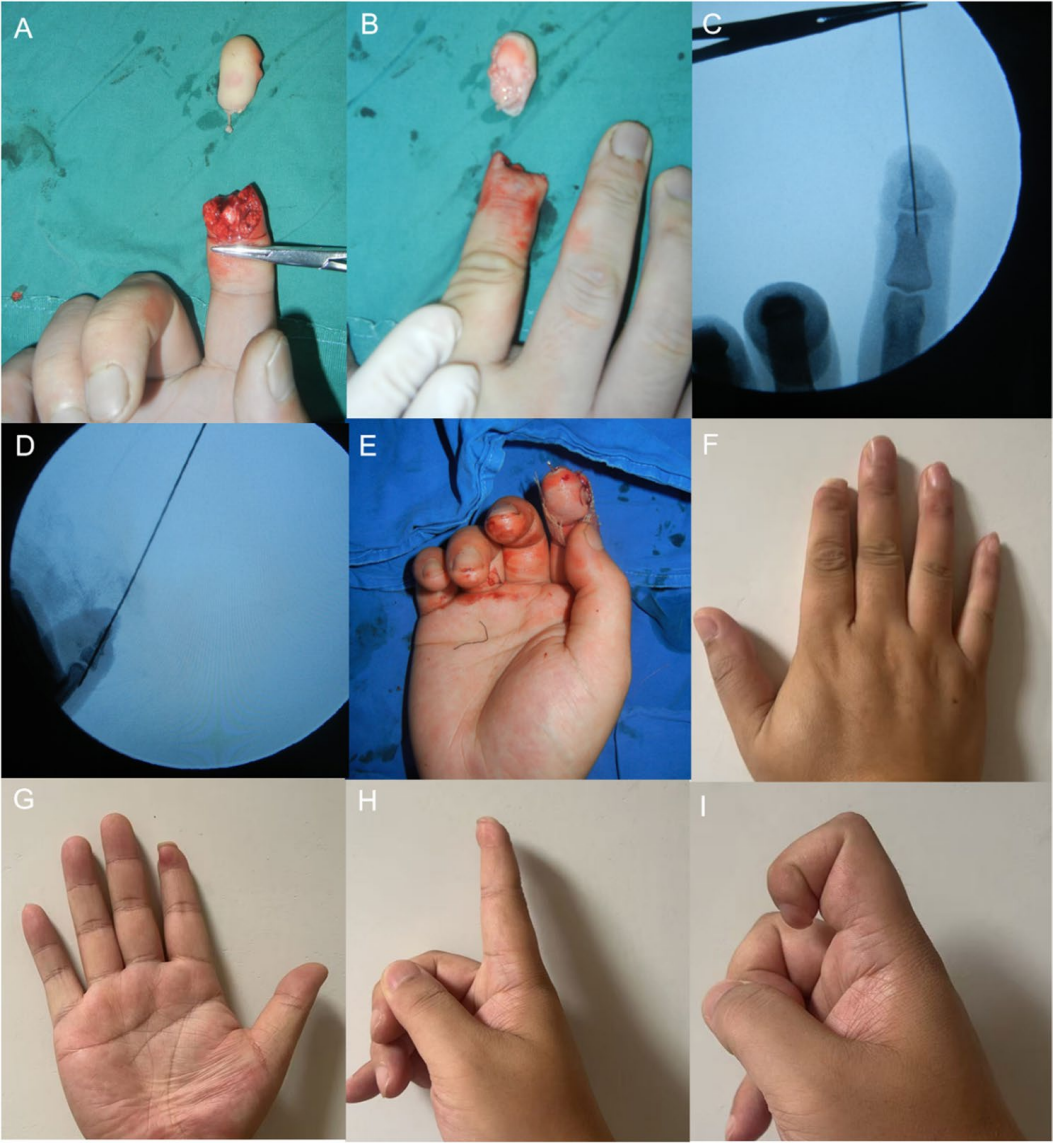
A young man underwent replantation for an avulsion-type amputation distal to the middle portion of the distal phalanx of the right index finger. **A-B**: Avulsion-type amputation distal to the middle portion of the distal phalanx of the right index finger. **C**-**D**: Fracture level and fixation. **E**: Immediate postoperative view showing restoration of blood supply. **F**-**G**: Mild atrophy of the replanted finger observed at follow-up, with acceptable appearance. **H**-**I**: Good flexion and extension function of the affected finger

**Fig. 4:**
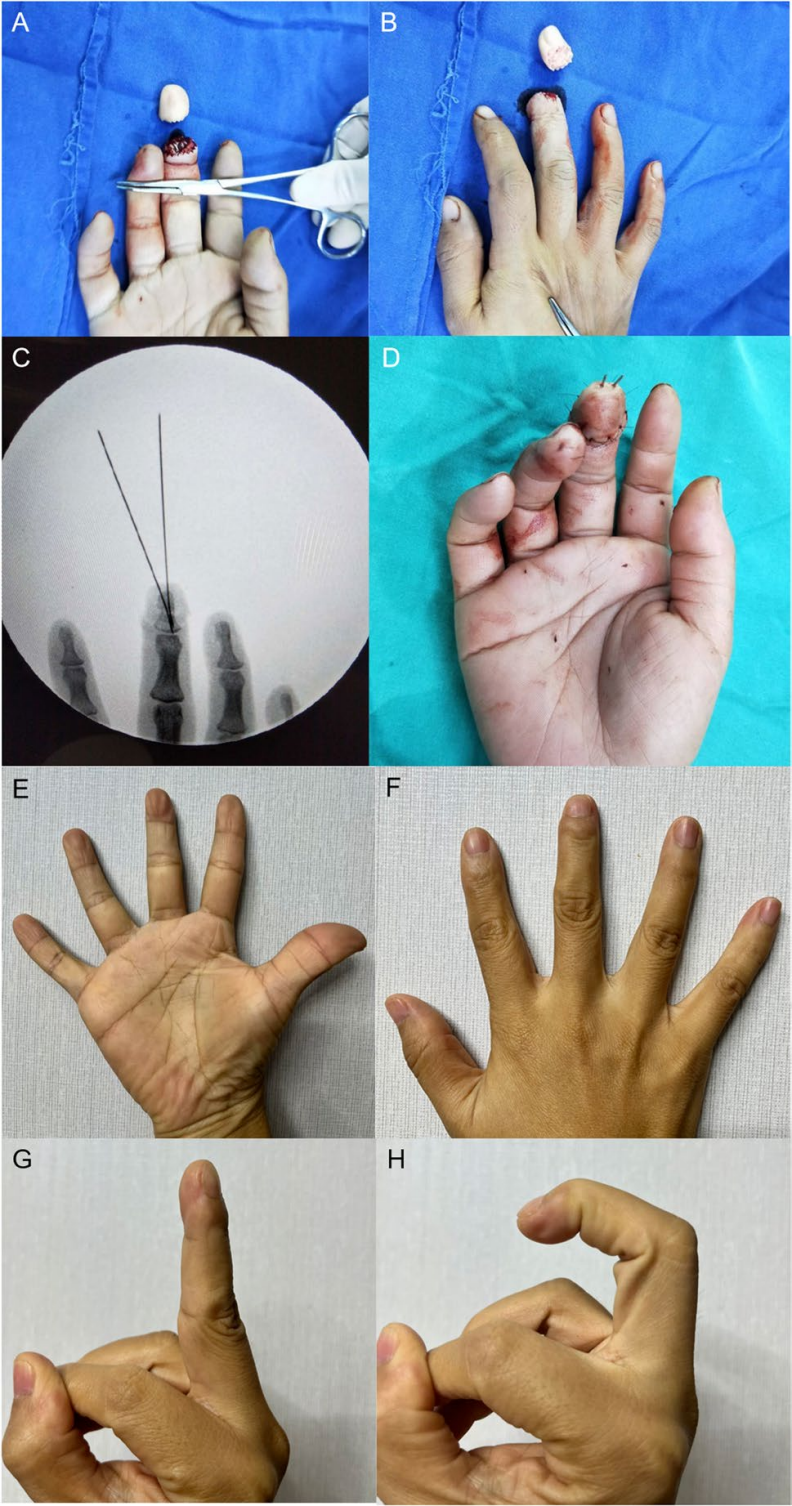
A young woman underwent replantation for an avulsion-type amputation distal to the middle portion of the distal phalanx of the right middle finger. **A**-**B**: Avulsion-type amputation distal to the middle portion of the distal phalanx of the right middle finger. **C**: Fracture level and fixation. **D**: Immediate postoperative view showing restoration of blood supply. **E-F**: Good appearance of the replanted finger observed at follow-up. **G**-**H**: Good flexion and extension function of the affected finger

**Fig. 5:**
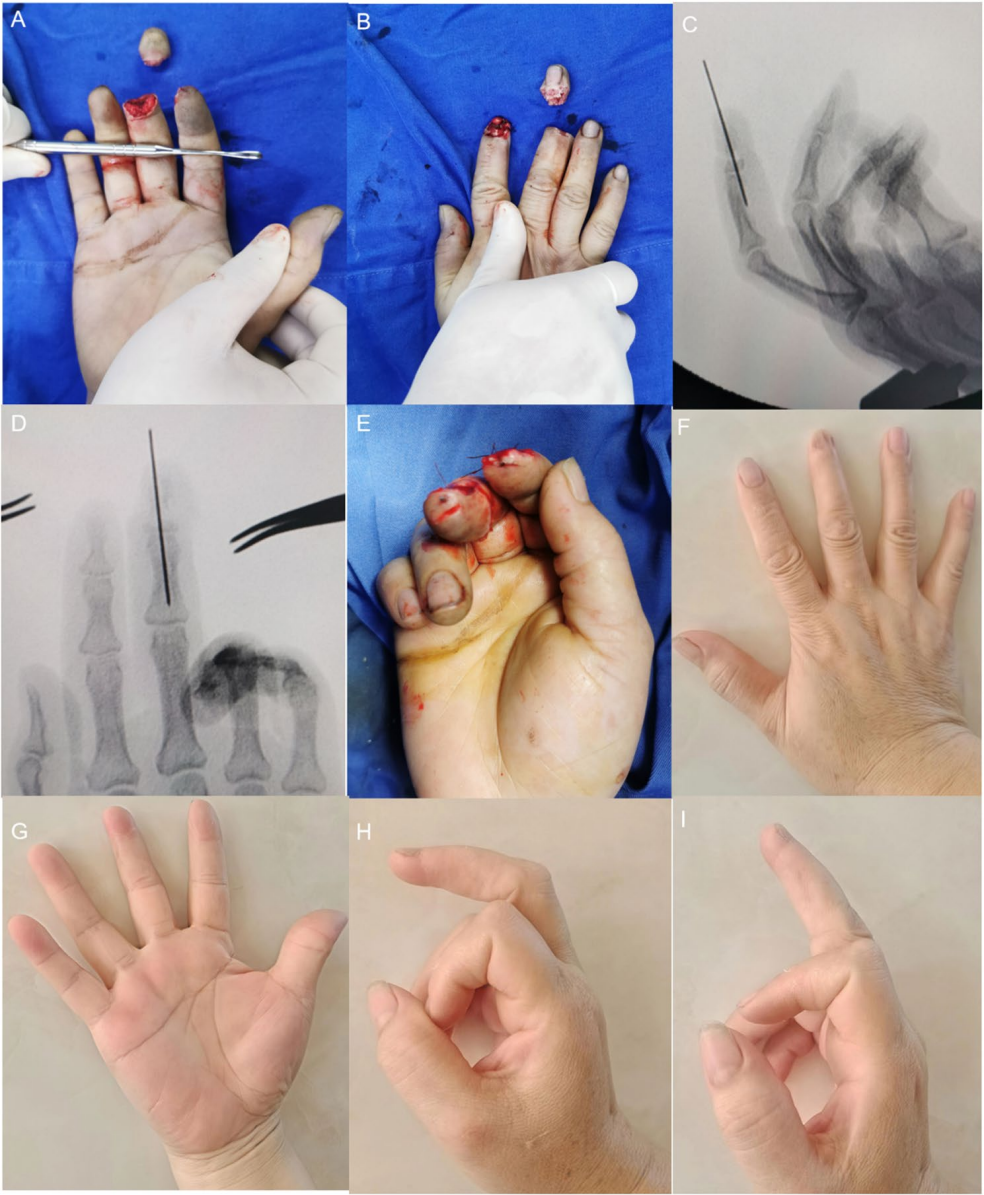
A middle-aged woman underwent replantation for an avulsion-type amputation distal to the middle portion of the distal phalanx of the right middle finger. **A-B**: Avulsion-type amputation distal to the middle portion of the distal phalanx of the right middle finger. **C**-**D**: Fracture level and fixation. **E**: Immediate postoperative view showing restoration of blood supply. **F**-**G**: Good appearance of the replanted finger observed at follow-up. **H**-**I**: The movement of the distal interphalangeal joint is slightly restricted

**Fig. 6:**
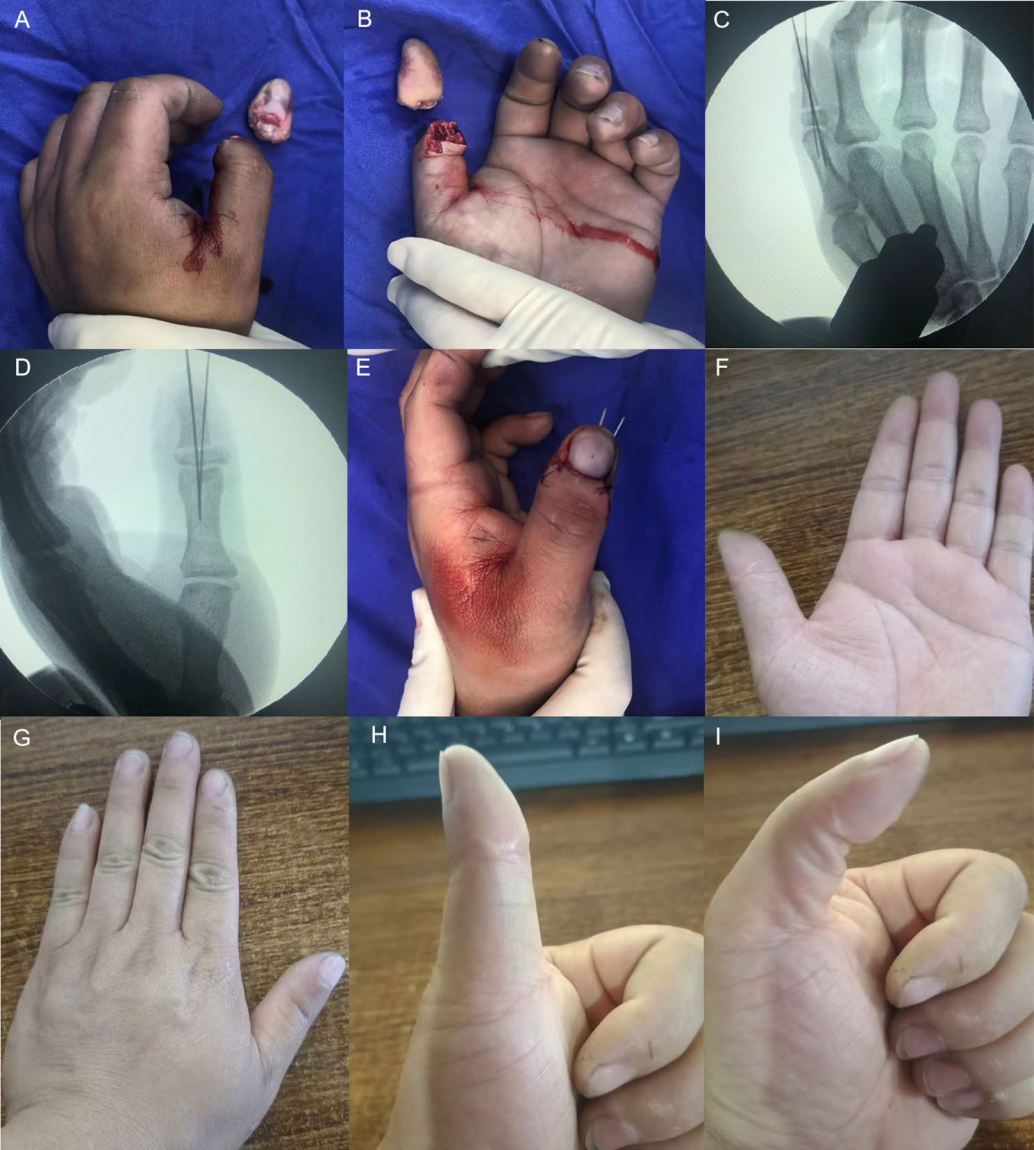
A young man underwent replantation for an avulsion-type amputation distal to the middle portion of the distal phalanx of the left thumb. **A-B** Avulsion-type amputation distal to the middle portion of the distal phalanx of the left thumb. **C**-**D** Fracture level and fixation. **E** Immediate postoperative view showing restoration of blood supply. **F-G** Good appearance of the replanted finger observed at follow-up. **H-I **The movement of the interphalangeal joint is slightly restricted

**Fig. 7:**
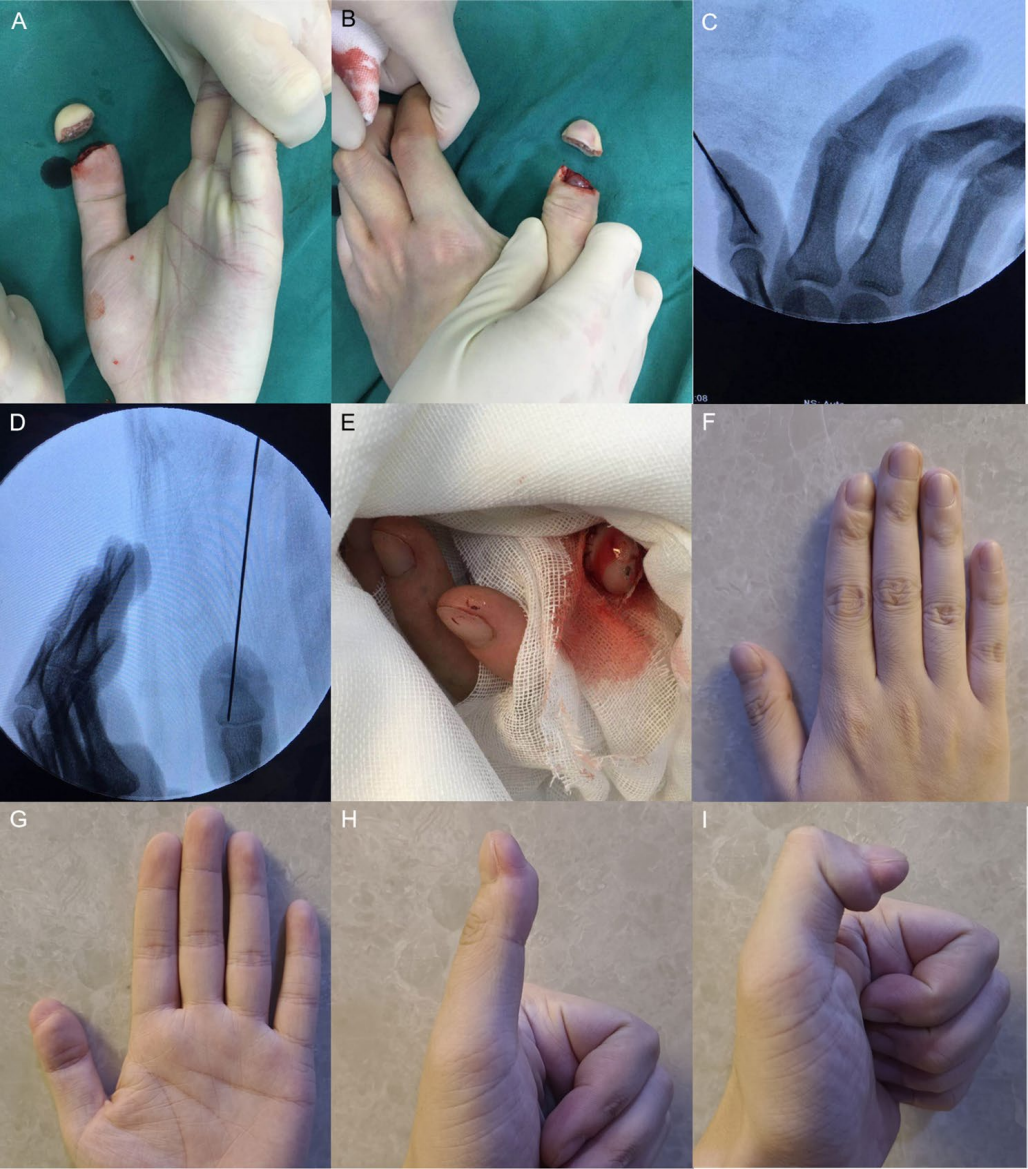
A young man underwent replantation for a clean-cut amputation distal to the distal portion of the distal phalanx of the left thumb. **A-B** Clean-cut amputation distal to the distal portion of the distal phalanx of the left thumb. **C**-**D** Fracture level and fixation. **E** Immediate postoperative view showing restoration of blood supply. **F**-**G** Mild atrophy of the replanted finger observed at follow-up, with acceptable appearance. **H-I** Good flexion and extension function of the affected finger

Typical Cases A, B, C, and D of Group 1 are shown in Figs. 3, 4, 5 and 6, respectively. Typical Case A of Group 2 is shown in Fig. 7. Comparisons between the two groups are presented in Table 2.

**Table 2:** Comparative analysis of outcome measures between the two patient groups

Outcome Measure	Avulsion Amputation	Clean-cut Amputation	*p*-value (95% CI)	Analysis of Main Causes
Survival Rate	57.10%	76.00%	0.006 (Group 1, OR = 0.672, 95% CI: 0.515 to 0.877; Group 2, OR = 1.599, 95% CI: 1.114 to 2.296)	1. More severe arterial contusion in Group 1 increased the risk of thrombosis.
				2. More severe soft tissue contusion at the amputation stump in Group 1 resulted in slower formation of granulation tissue bridges.
Risk of Vascular Crises	66/98	48/96	0.019 (Group 1, OR = 1.447, 95% CI: 1.061 to 1.975; Group 2, OR = 0.702, 95% CI: 0.530 to 0.928)	1. After arterial-only anastomosis, both groups had a high incidence of postoperative venous crises.
				2. Group 1 exhibited more severe arterial contusion and a higher incidence of arterial crises.
Sensory Recovery Score, median (interquartile range)	16 (16, 20)	16 (16, 20)	0.546 (Monte Carlo two-tailed *p* = 0.570, 95% CI: 0.560 to 0.579)	Less tissue at the fingertip resulted in decreased nerve regeneration distance.
Joint Range of Motion Score, median (interquartile range)	17.5 (16.25, 18.75)	18 (16, 19)	0.303 (Monte Carlo two-tailed *p* = 0.309, 95% CI: 0.300 to 0.318)	Injuries distal to the middle portion of the distal phalanx did not involve the interphalangeal joint, metacarpophalangeal joint or the tendons.
Appearance Score, median (interquartile range)	16 (16, 20)	16 (16, 20)	0.375 (Monte Carlo two-tailed *p* = 0.388, 95% CI: 0.379 to 0.398)	1. Group 1 had more severe tissue damage, greater shortening, mild atrophy, hyperpigmentation, and more scarring.
				2. Digits with prolonged vascular crises tended to have more severe atrophy.

## Discussion

Distal phalanx amputations of the fingers, particularly those occurring distal to the whorl of the volar pulp, exhibit anatomical characteristics that often permit only arterial anastomosis during replantation [6]. Although arterial perfusion is generally adequate after replantation, the absence of venous drainage disrupts vascular balance, causes congestion, and lowers the survival rate of replanted digits. Multiple factors influence the success of arterial-only anastomosis in distal phalanx replantation. A key determinant of tissue healing appears to be the early formation of granulation tissue bridging the amputation site. Neovascularisation within this granulation tissue establishes a pathway for venous drainage, restores vascular balance, and contributes to the survival of replanted digits. However, published evidence specifically addressing this mechanism remains limited.

Our clinical observations indicate that the absence of necrosis within the first postoperative week generally predicts the survival of replanted digits. During this period, the colour and capillary refill of the replanted digits typically return to normal. We hypothesised that this improvement corresponds to the completion of granulation tissue bridging at the amputation site, which typically occurs approximately 1 week postoperatively. We hypothesised that the more extensive soft tissue contusion in the avulsion group would require a longer time for granulation tissue bridging; this might partially explain the lower survival rate in this group than in the clean-cut group. Accordingly, during the replantation of avulsion amputations, we conducted a detailed assessment of soft-tissue contusion and performed more extensive debridement of devitalised tissue. This approach reduced the time needed for postoperative granulation tissue bridging and modestly improved survival.

Digital amputations distal to the middle portion of the distal phalanx do not involve key structures, such as joints or tendons, responsible for finger movement. Therefore, successful replantation generally leads to the favourable recovery of motor function [7]. Wong et al. reported that, even without nerve repair, skin two-point discrimination can recover substantially after distal phalanx replantation [8]. From an Asian perspective of aesthetics, the partial loss of a finger is often regarded as a disability and a form of bodily imperfection. Consequently, patients with amputations distal to the middle portion of the distal phalanx typically have a strong desire for replantation to preserve finger integrity, and report a high level of postoperative satisfaction. Hence, this type of finger amputation should be actively considered for replantation in clinical practice. Here, both groups achieved satisfactory outcomes in sensory recovery, joint range of motion, and appearance of replanted digits, while patient satisfaction was particularly high among younger and unmarried patients.

The distal phalanx has a small tissue volume and relatively low metabolic demand. Hence, it is theoretically less dependent on venous drainage than more proximal phalanges, which provides a physiological basis for the arterial-only anastomosis approach. Arterial patency is a prerequisite for the survival of replanted digits. We found that the two types of amputations distal to the middle portion of the distal phalanx showed a large disparity in the degree of arterial contusion, with differences in the likelihood of arterial spasm or thrombosis after arterial anastomosis. Consequently, the incidence of arterial crises and, ultimately, the survival rates differed between the two groups. Intraoperatively, we observed that in clean-cut digital amputations, the arterial contusion length was short, and excision of the contused segment resulted in a relatively low risk of thrombosis. Conversely, in avulsion amputations of the digit, the length of arterial contusion was longer. Our strategy involved meticulous microscopic examination of the arterial ends. The extent of debridement was determined by resection of the vessel back to a point where the intima appeared smooth and undamaged under high magnification, and where a ‘spurt test’ (observation of vigorous pulsatile bleeding after release of the proximal clamp) indicated good inflow. If the entire contused arterial segment were excised, direct anastomosis would be performed under significant tension. If superficial vein grafting was intended to repair the digital artery after excision, the extremely small vessel diameter distal to the middle portion of the distal phalanx—especially at or beyond the digital arterial arch—would greatly increase the technical difficulty of vascular grafting procedures, and would not guarantee anastomotic potency. Therefore, vascular grafting for arterial reconstruction is generally not suitable for fingertip amputations.

Some patients first developed venous crises, followed by arterial crises. This progression results from worsening venous congestion, which is usually indicative of a poor prognosis. When venous drainage is obstructed, congestion involves the entire venous system and extends to the capillary network, resulting in a marked increase in intravascular pressure and ultimately forcing cessation of arterial inflow. Prompt intermittent bleeding at the fingertip incision helps prevent this scenario by exchanging stagnant blood in the digit with fresh blood, thereby buying time for granulation tissue to bridge the amputation stump.

Surgical techniques that increase the contact surface for granulation tissue growth during digital replantation distal to the middle portion of the distal phalanx may help improve the survival rate of the amputated part. Puhaindran et al. evaluated outcomes of digital replantation with replanted digits buried under a palmar skin flap (dermal pocketing) and reported an 85% success rate [9]. These favourable outcomes may be attributed to the additional nutrients provided by the flap and to Neovascularisation within the newly formed granulation tissue, which improves venous drainage of the digit. Nevertheless, the use of pedicled palmar or abdominal flaps to pocket replanted digits often leads to complications such as joint stiffness, which limits the widespread application of this method. In contrast, arterial-only anastomosis with intermittent bleeding is a single-stage procedure that preserves joint mobility and avoids donor site morbidity, which may be preferable despite the need for intensive postoperative monitoring.

### Study limitations

This study adopted a single-centre retrospective design, which may have introduced certain limitations. First, some patients contributed data from more than one replanted digit, and this potentially introduced a clustering effect that was not adjusted for using multilevel modeling in our statistical analysis. Future prospective studies should account for this factor. Second, our reported survival rate applies to a selected cohort where arterial anastomosis was feasible. Third, our assessment criteria, although comprehensive for general digit replantation, may not fully address the specific outcomes of fingertip replantation. Future studies should develop and validate a dedicated scale that emphasises aesthetic evaluation and incorporates patient-reported satisfaction.

## Conclusion

In patients with fingertip avulsion amputations who receive arterial-only anastomosis without venous anastomosis, the survival rate of replanted digits, although lower than that achieved with conventional arterial and venous anastomosis, still exceeds 50%. Since this amputation type does not involve tendons or joints, satisfactory functional recovery and favourable cosmetic outcomes can be achieved. Therefore, arterial-only replantation represents a feasible and valuable alternative for complex fingertip avulsions where venous repair is not possible, offering a worthwhile attempt at digital salvage in selected cases. Furthermore, for Asian populations in which finger integrity is closely tied to personal identity and social perception, successful replantation provides significant psychological and aesthetic benefits that support its active consideration in clinical practice.

## Supplementary Information

Supplementary Material 1.

## Data Availability

The datasets used and/or analysed during the current study are available from the corresponding author on reasonable request.
