# Supplementary material for: Arterial-only anastomosis for fingertip avulsion amputations: survival rates and functional outcomes in a retrospective study of 194 digits
Source: BMC Surg. 2026 Mar 26;26:309. doi: 10.1186/s12893-026-03692-8 (PMC13141435; doi:10.1186/s12893-026-03692-8)
Supplement: Supplementary file 1 — Supplementary Material 1. [file 12893_2026_3692_MOESM1_ESM.docx]

**Additional file 1.**

**Provisional criteria for the functional assessment of digit replantation by the Chinese Society of Hand Surgery, Chinese Medical Association**

**1. Motor Function**

Assessed by total active motion (TAM) (20 Points)

1. Thumb: A. Thumb opposition (10 points)

Able: 10 points

Difficult: 5 points

Unable: 0 points

B. Active range of motion (ROM) of thumb joints (10 points)

Total ROM = ROM of the metacarpophalangeal joint + ROM of the interphalangeal joint

Total ROM > 90°: 10 points

≤ 90°: 5 points

Ankylosis: 0 points

1. Fingers: Active ROM of joints (20 points)

Total TAM = flexion of metacarpophalangeal joint + flexion of proximal interphalangeal joint + flexion of distal interphalangeal joint – total extension deficit

Total TAM 260°–200°: 20–16 points

190°–130°: 15–11 points

130°–100°: 10–6 points

<100°: 5–0 points

**2. Activities of daily living (ADL)** (20 points)

1) Picking up a needle (using the nail)

2) Picking up a coin (using the volar pulp)

3) Writing (three-finger pinch)

4) Lifting (e.g., suitcase handle, kettle handle and other heavy objects)

5) Holding a large cup (grip)

6) Hammering a nail (power grip)

7) Turning a screw (central grip)

8) Tying shoelaces (fine motor skills)

9) Buttoning (fine motor skills)

10) Opening a wide-mouth bottle (combination of power and precision grip)

Scoring for each item:

Completed well: 2 points

Completed but with poor movement: 1 point

Unable to complete: 0 points

**3. Sensory Recovery** (20 points)

British Medical Research Council (1954) grading criteria:

|  | Grade | Score |
| --- | --- | --- |
| S4 | Normal sensation, two-point discrimination <6 mm | 20 points |
| S3+ | Same as S3, but with residual two-point discrimination | 16 points |
| S3 | Complete recovery of superficial pain and tactile sensation, with no hypersensitivity | 12 points |
| S2 | Partial recovery of superficial sensation and tactile sensation | 8 points |
| S1 | Recovery of deep pain sensation in the skin | 4 points |
| S0 | No sensation in the nerve distribution area | 0 points |

**4. Blood Circulation Status** (10 points)

|  | Grade | Score |
| --- | --- | --- |
| Excellent | Normal skin colour and temperature, no special protection required | 10 points |
| Good | Slight skin discoloration, slightly lower skin temperature, sensitive to cold | 8 points |
| Fair | Pale or cyanotic skin colour, obviously lower skin temperature, and pronounced intolerance to cold | 4 points |
| Poor | Dusky or cyanotic skin colour, and intolerance to cold exposure | 2 points |

**5. Appearance** (20 points)

|  | Grade | Score |
| --- | --- | --- |
| Excellent | Replanted digit shows no rotation or non-functional angular deformity, normal contour, shortening <1 cm, and no significant functional impact | 20 points |
| Good | Replanted digit shows mild rotation or non-functional angular deformity, mild atrophy, shortening <1.5 cm, and no significant functional impact | 16 points |
| Fair | Rotation or angular deformity affecting function, atrophy, and shortening ≤2 cm | 8 points |
| Poor | Obvious deformity, shortening >2 cm, and severe impairment of function and appearance | 4 points |

**6. Return-to-Work Status** (10 points)

|  | Grade | Score |
| --- | --- | --- |
| Excellent | Resumed original work | 10 points |
| Good | Engaged in light work | 7 points |
| Fair | Unable to work but able to live independently | 3 points |
| Poor | Unable to work or live independently | 0 points |

Based on the six criteria above, the total score is graded as follows:

Excellent: 100–80 points

Good: 79–60 points

Fair: 59–40 points

Poor: <40 points

Notes:

1. For multiple-digit amputations, each joint of each digit is assessed separately; the scores are then summed and divided by the number of digits to obtain the average.

2. TAM: Total Active Motion.

3. ADL: Activities of Daily Living.
